# Repair of osteochondral defects with *in vitro* engineered cartilage based on autologous bone marrow stromal cells in a swine model

**DOI:** 10.1038/srep40489

**Published:** 2017-01-13

**Authors:** Aijuan He, Lina Liu, Xusong Luo, Yu Liu, Yi Liu, Fangjun Liu, Xiaoyun Wang, Zhiyong Zhang, Wenjie Zhang, Wei Liu, Yilin Cao, Guangdong Zhou

**Affiliations:** 1 Department of Plastic and Reconstructive Surgery, Shanghai 9th People’s Hospital, Shanghai Jiao Tong University School of Medicine, Shanghai Key Laboratory of Tissue Engineering, Shanghai, P.R. China; 2 National Tissue Engineering Center of China, Shanghai, P.R. China; 3 Research Institute of Plastic Surgery, Wei Fang Medical College, Wei Fang, Shandong, China; 4 Department of General Surgery, Wu Jing Hospital, Minhang District, Shanghai, China

## Abstract

Functional reconstruction of large osteochondral defects is always a major challenge in articular surgery. Some studies have reported the feasibility of repairing articular osteochondral defects using bone marrow stromal cells (BMSCs) and biodegradable scaffolds. However, no significant breakthroughs have been achieved in clinical translation due to the instability of *in vivo* cartilage regeneration based on direct cell-scaffold construct implantation. To overcome the disadvantages of direct cell-scaffold construct implantation, the current study proposed an *in vitro* cartilage regeneration strategy, providing relatively mature cartilage-like tissue with superior mechanical properties. Our strategy involved *in vitro* cartilage engineering, repair of osteochondral defects, and evaluation of *in vivo* repair efficacy. The results demonstrated that BMSC engineered cartilage *in vitro* (BEC-vitro) presented a time-depended maturation process. The implantation of BEC-vitro alone could successfully realize tissue-specific repair of osteochondral defects with both cartilage and subchondral bone. Furthermore, the maturity level of BEC-vitro had significant influence on the repaired results. These results indicated that *in vitro* cartilage regeneration using BMSCs is a promising strategy for functional reconstruction of osteochondral defect, thus promoting the clinical translation of cartilage regeneration techniques incorporating BMSCs.

Functional repair of large osteochondral defects is always a great challenge in orthopaedic surgery because of complex osteochondral structure and the limited regeneration ability of cartilage^1^. Tissue engineering, which can regenerate live and functional tissue similar to native tissue, may provide a promising strategy^2^,^3^. In fact, autologous chondrocyte implantation/transplantation (ACI/ACT) has been approved by the United States Food and Drug Administration for clinical treatment of articular cartilage defects^4^,^5^,^6^. However, strategies using chondrocytes as a cell source will inevitably be associated with limited cell supply, donor site morbidity, and most importantly, restoration of only the cartilage layer but not underlying subchondral bone^7^,^8^,^9^. Therefore, identification of a more appropriate cell source to promote regeneration of both cartilage and subchondral bone is an urgent issue.

Bone marrow stromal cells (BMSCs) are considered an ideal cell source for osteochondral regeneration because of insignificant donor-site morbidity, robust proliferative capacity, and committed potentials for both cartilage and bone^10^,^11^,^12^. Many studies, including our previous investigations, have already demonstrated that BMSCs could repair osteochondral defects with both regenerated cartilage and bone under the regulation of articular osteochondral microenvironments^1^,^13^,^14^. However, most of these reports were based on cell-scaffold constructs as implants, which have several disadvantages for both surgical manipulation and tissue regeneration, such as inconvenient handling for surgeons, cell leakage^15^, inflammatory reaction triggered by abundant un-degraded scaffolds^16^,^17^, difficulty in quality control prior to implantation, insufficient mechanical properties, and spontaneous differentiation within the traumatic environment after implantation^18^,^19^. Therefore, the total success rate of osteochondral defect repair is not satisfactory^20^, which obviously limits further clinical translation of cell-scaffold constructs.

We propose that *in vitro* cartilage regeneration is the key to solve these problems. There are many advantages of *in vitro* cartilage regeneration compared with *in vivo* chondrogenesis^21^, especially for stem cell-based cartilage regeneration. Primary advantages include convenient handling for surgeons (similar to autologous cartilage transplantation), reduced cell leakage^15^, mild inflammatory reaction because of minimal or no remnant scaffold^16^,^17^, convenient quality control before implantation, superior mechanical properties, and more reliable cartilage regeneration after implantation (cartilage had formed before implantation and, thus, was less influenced by the traumatic environment)^18^,^19^. Despite these advantages, some important issues are still unknown. First, what is the cartilage formation process for BMSC *in vitro* engineered cartilage (BEC-vitro)? Second, can implantation of BEC-vitro alone realize tissue-specific repair of articular osteochondral defects with both cartilage and subchondral bone in a large animal model? Third, does the maturity level of BEC-vitro affect the efficacy of repair? And finally, whether prolonged *in vitro* pre-culture has influence on *in situ* integration of the implant? All these issues directly restrict the clinical translation of BEC-vitro and, thus, require thorough investigation.

To address these issues in this study, hybrid pigs, whose knee joints were very close to human’s ones in structure and load condition, were employed as an animal model. Autologous BMSCs were seeded into polyglycolic acid/polylactic acid (PGA/PLA) scaffolds, and were chondrogenically induced for 2–12 weeks. The cartilage formation process and the hypertrophic character of BEC-vitro were investigated at different time points. Based on this, *in vitro* engineered constructs at 2, 4, and 8 weeks were used to repair autologous articular osteochondral defects, in order to clarify the feasibility, superiority, and optimal implantation timeline of BEC-vitro for repairing articular osteochondral defects. The current study provides detailed insights for future clinical applications of *in vitro* engineered cartilage incorporating BMSCs.

## Results

### Extracellular matrix (ECM) production by BMSCs on PGA/PLA scaffolds

**Figure 1:**
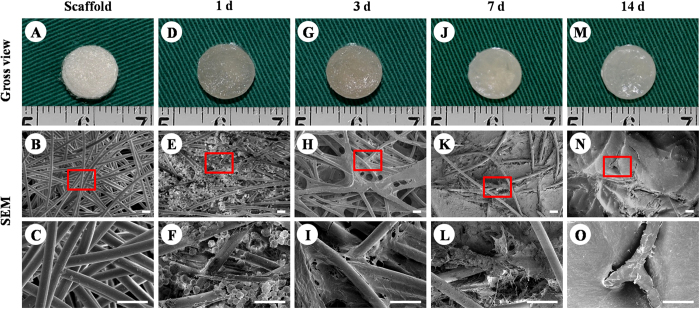
ECM production by BMSCs on PGA/PLA scaffolds. Unwoven PGA fibres were compressed to form a cylindrical-shaped scaffold with porous structure (**A**–**C**). BMSCs on the scaffold display round shapes within 24 h and distribute throughout PGA fibres (**D**–**F**). Three days after cell seeding, BMSCs started to spread and connect to PGA/PLA fibres with little ECM production (**G**–**I**). At 7 days, BMSCs produced enough ECM to wrap around PGA fibres and cover the porous structure (**J**–**L**). At 2 weeks, constructs presented an ivory-white appearance and the PGA fibres had been completely covered by abundant ECM (M–O). Scale bar = 50 μm.

ECM production by BMSCs on PGA/PLA scaffold was first evaluated by scanning electron microscopy (SEM) during the early stage of *in vitro* culture. PGA/PLA scaffolds maintained a cylindrical shape with porous structures (Fig. 1A–C). After cell seeding, the constructs still maintained their original shape and size, and gradually presented an ivory-white appearance with the culture time (Fig. 1D,G,J,M). SEM showed BMSCs on PGA fibres exhibited round shapes within 24 hours (Fig. 1E,F), and then, with increased ECM production, gradually spread to wrap around the PGA fibres as *in vitro* culture time progressed (Fig. 1H,I,K,L,N,O). Collectively, these results indicated that BMSCs maintained good ECM production ability on PGA/PLA scaffolds.

### Gross and histological evaluation of BEC-vitro

**Figure 2:**
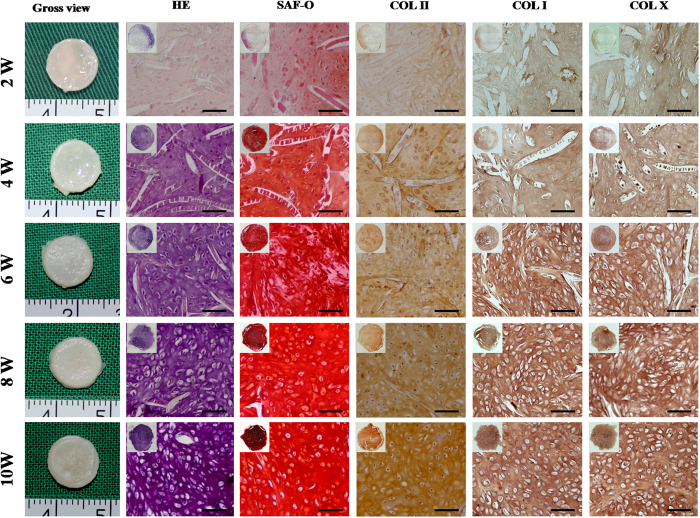
Gross view and histology of *in vitro* BMSC cartilage formation. *In vitro* chondrogenesis and maturation of BMSCs represented a time-dependent manner. At 2 weeks, constructs started to form cartilage-like tissue at the edge with ECM deposition; at 4 weeks, the constructs basically formed homogeneous, cartilage-like tissue with uniform ECM deposition and un-degraded PGA fibres; at 6 weeks, newly formed cartilage became more mature with typical lacuna structures and less residual PGA; at 8 and 10 weeks, samples became mature, homogeneous cartilage-like tissue with abundant lacuna structures and strongly positive staining for cartilage-specific matrices. Notably, expression of COL I and COL X was detected in all samples at different time points. Scale bar = 100 μm.

The quality of *in vitro* cartilage regeneration and its potential of endochondral ossification are key factors influencing the efficacy of osteochondral defect repair^22^. Therefore, gross and histological evaluations of BEC-vitro were first performed to investigate *in vitro* cartilage formation and its hypertrophic character. With increased *in vitro* induction time, the constructs gradually presented a cartilaginous ivory-white appearance (Fig. 2). Histological examination showed that, with increased *in vitro* culture time, constructs gradually displayed mature cartilage features with typical lacuna structures, increased ECM deposition, and strongly positive staining of cartilage-specific matrices, such as sulfated glycosaminoglycan (GAG) and collagen type II (COL II; Fig. 2). Generally, preliminarily cartilage formation occurred at 4 weeks and achieved a mature and homogeneous state by 8 weeks, indicating a time-dependent trend of *in vitro* chondrogenesis and maturation (Fig. 2).

It was worth noticing that expression of the hypertrophy-related proteins such as collagen type I and X (COL I, COL X) was detected in all BEC-vitro samples at different time points, indicating that BEC-vitro maintained endochondral ossification potential even under a chondrogenic culture system (Fig. 2). In addition, residual PGA fibres showed a decreased trend for both number and length with increased *in vitro* culture time (Supplementary Fig. 1).

### Biomechanical and biochemical evaluations of BEC-vitro

**Figure 3:**
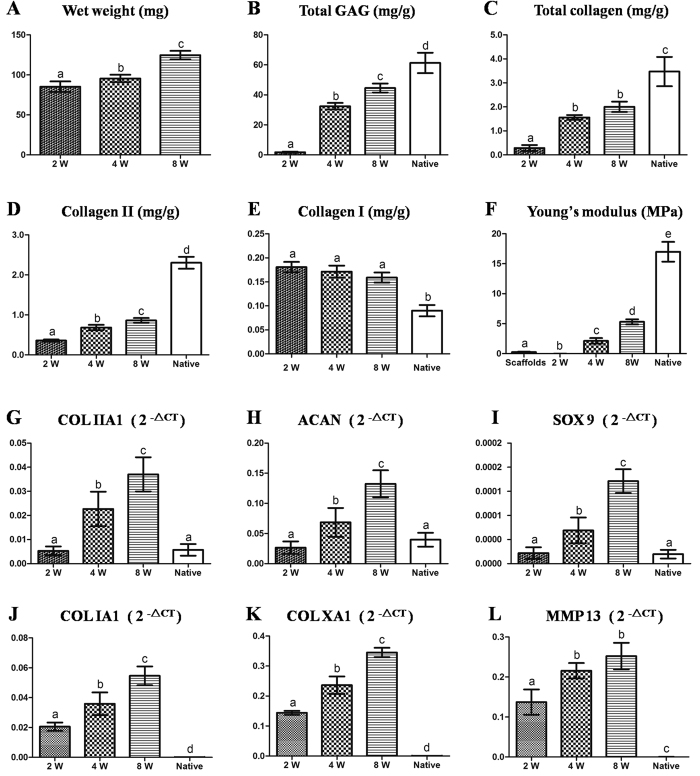
Biochemical, biomechanical and cartilage-related gene analyses of *in vitro* engineered tissues at various time points. All examinations of wet weight (**A**), GAG (**B**) contents, total collagen (**C**), total collagen II (**D**), Young’s modulus (**F**), expressions of cartilage-related genes (**G**–**I**), and hypertrophy-related genes (**J**–**L**), except for collagen I content (**E**), showed an increasing trend with *in vitro* induction time. Expression of cartilage-related genes in samples at 4 and 8 weeks were even higher than those found in native cartilage. The columns with different letters indicate statistical significance.

Biomechanical and biochemical evaluations of BEC-vitro further confirmed the above observations (Fig. 3). PGA/PLA scaffolds alone and BEC-vitro constructs at 2-week group showed poor mechanical strength (Supplementary Videos 1–2; Fig. 3F). BEC-vitro exhibited increased mechanical strength with good elasticity at 4 weeks, and achieved much better mechanical properties at 8 weeks (Supplementary Videos 3–4; Fig. 3F). All quantitative examinations related to cartilage maturity level, such as wet weight, contents of cartilage ECM (total GAG, total collagen, and COL II), and Young’s modulus, significantly increased with *in vitro* induction time (Fig. 3A–D,F; *p* < 0.05). However, COL I content didn’t showed significant increased during *in vitro* culture (Fig. 3E; p > 0.05). These results indicated that *in vitro* chondrogenic induction could significantly promote BMSCcartilage formation with increased culture time.

### Quantitative Real-Time polymerase chain reaction (QRT-PCR) analysis of BEC-vitro

Cartilage specific genes and hypertrophy-related genes were further analysed by qRT-PCR to evaluate the *in vitro* cartilage formation and its endochondral ossification potential at various time points. According to the current results, expression levels of cartilage specific genes COL IIA1, aggrecan (ACAN), and Sry related HMG box-9 (SOX 9) (Fig. 3G–I) rapidly increased with *in vitro* culture time and even achieved a much higher level than those found in native articular cartilage after 4 weeks (p < 0.05). Surprisingly, hypertrophy-related genes COL IA1, COL XA1, and matrix metal proteinase 13 (MMP13) also increased with *in vitro* culture time (Fig. 3J–L). These results indicated that *in vitro* chondrogenic induction not only promoted chondrogenic differentiation of BMSCs but also activated their endochondral ossification potential.

### Gross view and grading of repaired regions

**Figure 4:**
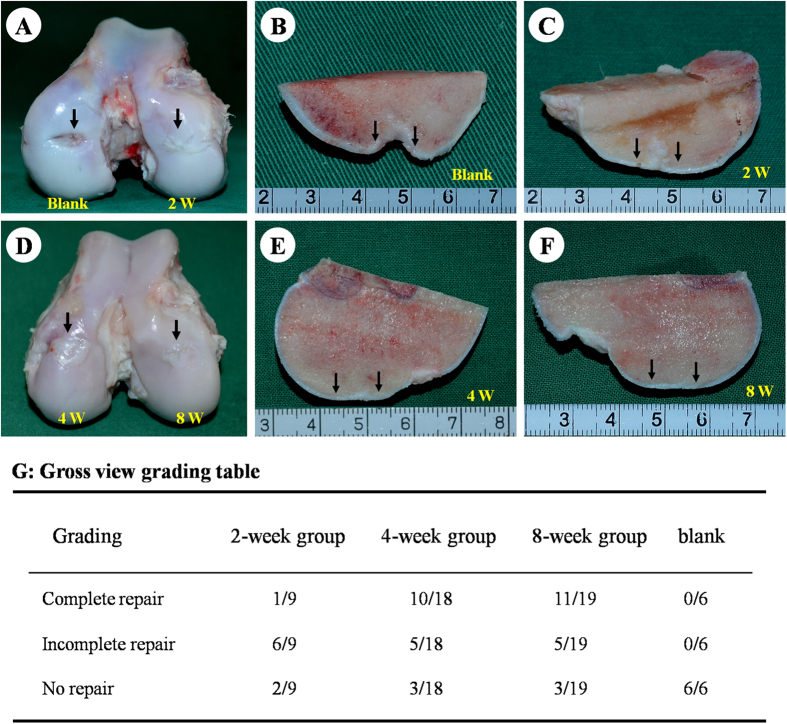
Gross view and grading of repaired regions. Most osteochondral defects in the 2-week group and all of the defects in the blank group were covered by fibrous-like tissue that infiltrated deep into subchondral bone; irregular surfaces were also observed (**A**–**C**). Defects in the 4- and 8-week groups were primarily repaired by glistening, white cartilage-like tissue and bone-like tissue exhibiting relatively smooth surfaces, good integration, and cartilage thickness similar to native structures (**D**–**F**). The detailed gross-view grading was listed in the grading table (**G**). Black arrows indicate repaired regions.

As shown in Fig. 4, most defects in the 2-week group were repaired by fibrous-like tissue deep into subchondral bone areas (Fig. 4A,C). In contrast, more than half of the defects in the 4- and 8-week groups were completely repaired by cartilage- and bone-like tissues (Fig. 4D–F,G; Supplementary Fig. 2). These results indicate prolonged *in vitro* chondrogenic induction of BMSCs may improve the efficacy of osteochondral defect repair. Noticeably, in contrast to 4- and 8-week groups, all defects in the blank group left obvious tissue defect or were mainly filled with fibrous tissues (Fig. 4A,B,G), implying the implanted BEC-vitro might directly participate in both articular cartilage and subchondral bone regeneration. Nevertheless, several defects in the 4- and 8-week groups still showed no repair, which may result from individual difference or some unclear negative factors (Fig. 4G).

### Histological examination of repaired regions

**Figure 5:**
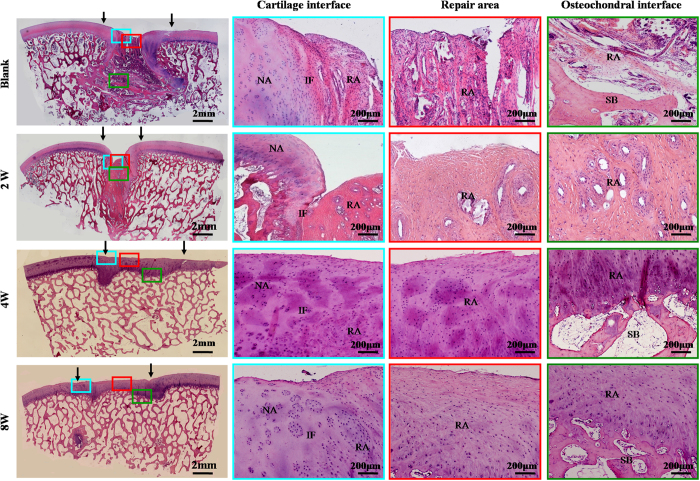
HE examination of repaired regions. Repaired tissues in the 2-week and blank groups showed signs of fibrogenesis and partial ossification with clear interface to the native tissue. Repaired tissues in the 4- and 8-week groups were primarily composed of cartilage- and bone-like tissues. Newly formed cartilage in these two groups exhibited a higher cell density than the surrounding native cartilage, with mature lacuna structures and satisfactory interface integration of both cartilage and osteochondral interfaces. NA: native area; IF: interface; RA: repaired area; SB: subchondral bone. Black arrows indicate repaired regions.

**Figure 6:**
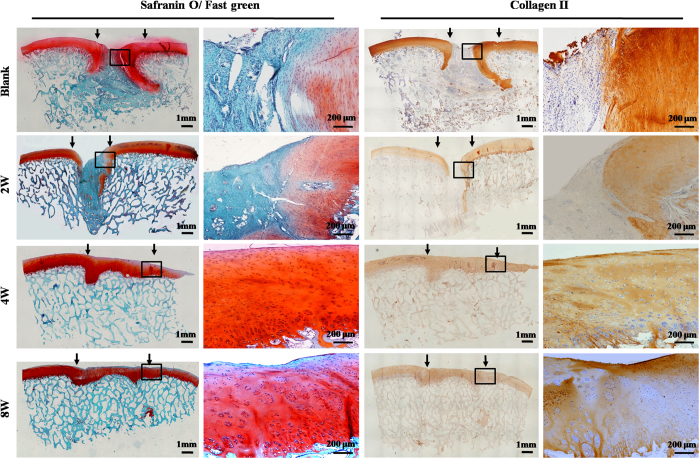
Cartilage-specific staining of repaired regions. Negative staining for Safranin-O and collagen type II was observed in the repaired tissue of the 2-week and blank groups. Repaired tissues in the cartilage defect regions of the 4- and 8-week groups showed continuous and strongly positive staining for Safranin-O and collagen II, but negative staining for these markers in bone defect regions. Black arrows indicate repaired regions.

**Figure 7:**
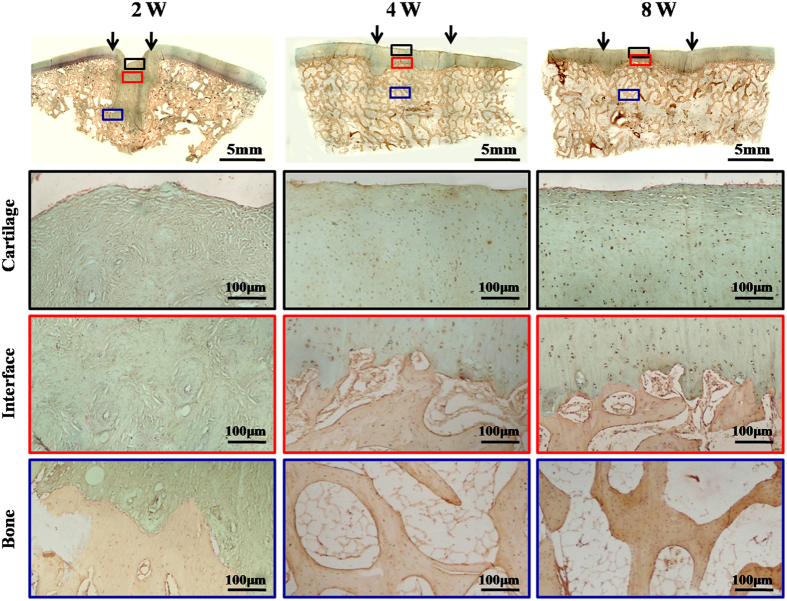
OCN immunohistochemical staining of repaired regions. In the 2-week group, the entire repaired region presented fibrous tissue with negative staining for OCN. In the 4- and 8-week groups, the repaired regions showed OCN-negative cartilaginous regions (black boxes) and OCN-positive subchondral bone regions (blue boxes) with distinct interfaces (red boxes), similar to the surrounding native structures. Black arrows indicate repaired regions.

Histological examinations further supported the above observations. Repaired regions in the 2-week and blank groups mainly consisted of fibrous tissue that was negative for GAG, COL II, and osteocalcin [(OCN), (Figs 5, 6 and 7)], and strongly positive for COL I (Supplementary Fig. 3). In contrast, repaired regions in the 4- and 8-week groups were repaired primarily by cartilage-like tissue and cancellous bone, with strong cartilage ECM staining in cartilage-repaired regions and strong bone-marker expression in bone-repaired regions, confirming the tissue-specific repair of osteochondral defects in these two groups (Figs 5, 6 and 7; Supplementary Figs 2–3). In addition, histological grading scores further demonstrated that the 4- and 8-week groups achieved much better repair than the 2-week and blank groups (Supplementary Table 1). Collectively, these results support the notion that sufficient *in vitro* chondrogenic induction of BMSCs was necessary to improve the efficacy of osteochondral defect repair. Nevertheless, interfaces between repaired and native cartilage in the 4- and 8-week groups could still be distinguished by differences in cartilage thickness, surface regularity, and cell density (Figs 5, 6 and 7; Supplementary Fig. 2). These results further implied that the implanted BEC-vitro might directly participate in cartilage regeneration. Furthermore, neocartilage in the central area of defects basically presented negative expression of collagen I, implying these newly formed cartilage primarily consisted of hyaline-like cartilage (Supplementary Fig. 3).

### Biomechanical and biochemical evaluations of the repaired tissues

**Figure 8:**
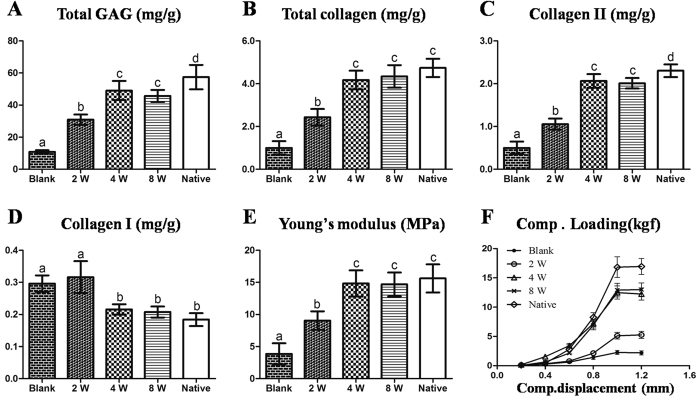
Biomechanical and biochemical evaluations of repaired tissue. Compared with the 2-week and blank groups, repaired tissues in the 4- and 8-week groups showed higher contents of GAG (**A**), total collagen (**B**) and collagen II (**C**), as well as increased Young’s moduli (**E**) but lower collagen I content (close to native group levels) (**D**). Compressive loading-displacement curves showed different trends among groups (**F**). No significant differences were observed in the above examinations between the 4- and 8-week groups. Columns with different letters indicate statistical significance.

In general, neocartilage in the 4- and 8-week groups showed higher cartilage ECM contents (GAG, total collagen, and collagen II) and stronger mechanical properties compared with the 2-week and blank groups (Fig. 8A–C,E,F; p < 0.05). Whereas, collagen I contents of neocartilage presented a contradictory trend amongst these groups, which was consistent with the histological examination (Fig. 8D; Supplementary Fig. 3). Noticeably, no significant differences were observed between the 4- and 8-week groups in any of the examinations described above, and all the examinations in these two groups were close to native cartilage (Fig. 8A–F). These results indicated repaired cartilage in the 4- and 8-week groups achieved levels similar to native articular cartilage with regard to both cartilage quality and function.

## Discussion

Although some studies have reported the feasibility of repairing articular osteochondral defects using BMSC-scaffold constructs^13^,^23^, no significant breakthroughs have been achieved in stable reparative outcomes because multiple disadvantages of cell-scaffolds have apparently impeded the clinical translation of this approach^1^,^17^,^24^. *In vitro* cartilage regeneration strategies may help to address this issue. The current study revealed that BMSC *in vitro* engineered cartilage presented a time-depended maturation process and confirmed that the implantation of BEC-vitro alone could successfully realize tissue-specific repair of osteochondral defects with both cartilage and subchondral bone. Furthermore, the maturity level of BEC-vitro had significant influence on the repaired results. These results suggest that BMSC *in vitro* engineered cartilage represents a promising strategy for clinical repair and recovery of osteochondral defects.

*In vitro* cartilage regeneration is the key point we proposed to avoid the disadvantages of direct cell-scaffold construct implantation. The current results showed that *in vitro* cartilage formation by BMSCs presented a gradually mature course with increased induction time and was accompanied by gradual degradation of scaffolds, which assisted in avoiding cell leakage^15^, inflammatory reactions triggered by scaffolds^16^,^17^, and spontaneous differentiation of BMSCs after implantation^18^,^19^. Furthermore, BEC-vitro achieved appropriate mechanical strength for surgical operation and was convenient for quality control measures before *in vivo* implantation, which facilitates more stable cartilage regeneration *in vivo* compared with cell-scaffold constructs. More importantly, high expressions of hypertrophy-related genes provided a probability for bone regeneration of BEC-vitro through endochondral ossification pathways.

Despite this speculation, whether BEC-vitro could actually repair articular osteochondral defect remained uncertain. The current results revealed that BEC-vitro could successfully repair articular osteochondral defects in both the 4- and 8-week groups with ideal interface integration between repaired and native tissues. Most importantly, the implantation of BEC-vitro alone could realise tissue-specific repair of osteochondral defects with surface cartilage and underlying cancellous bone in these two groups. In contrast, neither cartilage nor subchondral bone regeneration was observed in the blank group, implying that implanted BEC-vitro might directly participate in cartilage and subchondral bone regeneration.

Why implantation of BEC-vitro alone could realise regeneration of both cartilage and subchondral bone is an important issue. It is widely accepted that BMSCs exhibit multiple lineage potentials, such that their ultimate *in vivo* fate is primarily determined by the implanted microenvironment^25^,^26^. Under articular cartilage microenvironment, endogenous growth factors in synovial fluid, mechanical stimulation, and hypoxia environment were favourable factors for driving chondrogenic differentiation of the implanted BMSCs^27^,^28^,^29^. Therefore, it is reasonable that BEC-vitro formed hyaline cartilage-like tissue under articular cartilage microenvironment in the current study. The most concerned issue is why BEC-vitro transformed into subchondral bone under bone defect environment. The following two reasons might account for this issue: 1) BEC-vitro had strong potential of endochondral ossification. Our previous study has demonstrated that BEC-vitro, especially within 8 weeks, had a high-risk of ectopic ossification after implanted into subcutaneous environment^30^. The high expressions of hypertrophy-related genes at both protein and gene levels in the current study further confirmed the endochondral ossification potential of BEC-vitro (Figs 2, 3). (2) Regulation of osteogenic microenvironment. Once implanted into bone defect microenvironment, BEC-vitro might be stimulated by endogenous osteogenic factors, mechanical stimulation, and normoxia to transform into subchondral bone by activation of endochondral ossification pathways^31^,^32^. Although these factors provided reasonable explanations for the transform of BEC-vitro towards subchondral bone, the exact mechanisms still need to be further investigated.

Whether the maturity level of BEC-vitro affected the efficacy of osteochondral defect repair was another important issue. BMSC-scaffold constructs without adequate cartilage formation might only achieve limited cartilage regeneration because of a lack of sufficient *in vitro* chondrogenic induction and the influence of abundant un-degraded scaffolds^13^. However, fully mature engineered cartilage (similar to osteochondral arthroscopic grafting) might be difficult to integrate with surrounding native tissue and exhibit poor endochondral ossification potential^33^,^34^. Therefore, determining an optimal time for *in vivo* implantation was the key in achieving satisfactory results for both osteochondral tissue regeneration and interface healing. The current results showed that satisfactory tissue regeneration and interface healing were only achieved in the 4- and 8-week groups, but not in the 2-week group (mainly fibrous tissue formation). These results seemed to be inconsistent with the report of Miot S *et al*.^35^, which might be a result of different cell sources and scaffolds. For the current study, two key points might account for the failure observed in the 2-week group. First, BMSC-scaffold constructs at 2 weeks likely had difficulty developing into cartilage-like tissue after *in vivo* implantation because of a lack of sufficient chondrogenic factors and the negative influence of inflammatory cytokines in a surgical trauma niche^24^,^27^,^29^. Second, abundant remnant PGA fibres and their degradation products triggered serious inflammatory reactions, thus interrupting cartilage and bone regeneration *in vivo*^16^,^17^. In contrast, in the 4- and 8-week groups, BEC-vitro had basically formed cartilage-like tissues with less scaffold remnants, less dependence on extra growth factors, and stronger resistance to inflammatory cytokines in the trauma niche (protected by self-secreted ECM), and thus could proceed to develop into mature cartilage and subchondral bone in osteochondral microenvironments.

Although BEC-vitro presented successful repair results for articular osteochondral defects, the complete repair rate did not reach our expectations. Several factors might account for the unsatisfactory repair rate. First, large-size defects (10-mm diameter), which reached over 2/3 of the femoral condyle area in the current study (Supplementary Fig. 4B), might significantly increase the difficulty of defect repair due to serious destruction of the local microenvironment and less mechanical support from surrounding cartilage^36^,^37^. Second, severe surgical trauma caused by too many defects (4 defects) in both legs might have negatively influenced osteochondral regeneration and articular function recovery^37^,^38^. Third, appropriate postoperative care and rehabilitation, which is difficult to guarantee in a large animal model, might impose another important negative factor^39^,^40^,^41^. Finally, individual differences among animals might also influence ultimate repair outcomes. Besides, for future clinical translation, other concerned issues such as biosafety, *in vivo* long-term fate, and the feasibility of repairing larger osteochondral defects, still need to be further investigated.

## Methods

All procedures in the present study were strictly executed according to the regulations and laws of our country and in accordance with the Standing Committee on Ethics in China (The State Scientific and Technological Commission of China)^42^. Animal experiments were approved by the Shanghai Jiao Tong University Committee on Animal Care and were conducted by the Key Laboratory of Tissue Engineering at Shanghai Ninth People’s Hospital, affiliated with Shanghai Jiao Tong University in China. All experiments involving animals were performed in accordance with the approved guidelines.

### General experimental design

A total of sixteen 6-month-old hybrid pigs (7 male and 9 female) weighing 45–50 kg were used in this research (Shanghai Jiagan Biological Technology Co., Shanghai, China). General experimental procedures included: isolation and expansion of BMSCs, *in vitro* cartilage engineering, evaluations of *in vitro* cartilage formation, establishment of osteochondral defect repair model, and evaluations of *in vivo* repair efficacy. BEC-vitro at 4 and 8 weeks was implanted in the experimental groups (4- and 8-week groups respectively), BEC-vitro at 2 weeks as cell-scaffold construct control, while untreated osteochondral defects served as a blank control.

### Isolation and expansion of BMSCs

Bone marrow was aspirated from the anterior superior iliac spine of each animal. BMSCs were isolated, cultured and expanded in regular medium [Dulbecco’s Modified Eagle’s Medium (DMEM; Hyclone Laboratories, Logan, UT) containing 10% foetal bovine serum (FBS, Hyclone Laboratories, Victoria, Australia), and 1% Penicillin-streptomycin-amphotericin B (Hyclone Laboratories, Logan, UT) according to previously reported methods^13^,^43^. BMSCs were harvested at passage two for further experiments.

### *In vitro* cartilage engineering using BMSCs

Fifteen milligrams of unwoven PGA fibres (National Tissue Engineering Center of China, Shanghai, China) were prepared into cylinders with a 10-mm diameter and 2-mm height, and then 0.5% PLA (Sigma-Aldrich, St. Louis, MO) solution was added to solidify the shape of PGA scaffolds^30^,^44^. Passage two BMSCs were harvested and seeded into PGA/PLA scaffolds at a concentration of 60×10^6^ cells in 0.1 mL/scaffold, incubated for 4 h^45^, and then cultured in regular medium for 48 h. After that, culture medium was replaced with chondrogenic medium [DMEM supplemented with 10 ng/mL transforming growth factor β1 (TGF-β1, HumanZyme, Chicago, IL), 40 ng/mL dexamethasone (Sigma-Aldrich), 100 ng/mL insulin-like growth factor 1 (IGF-1, R&D Systems, Minneapolis, MN), and other addictives; Serum free], in which cells were cultured for 2–12 weeks.

### SEM analysis of BMSC-scaffold constructs

The cell-scaffold constructs at various time points *in vitro* culture (1, 3, 7, and 14 days, n = 3 for each time point) and PGA/PLA scaffolds (n = 3) were examined by SEM (Philips XL-30, Amsterdam, Netherlands) to evaluate attachment and ECM production of BMSCs on the scaffolds according to previously reported procedures^46^.

### Gross view and histological evaluation of BEC-vitro

At various time points from 2–10 weeks, BEC-vitro were grossly examined and immediately weighed. Next, samples were fixed (n = 3), embedded in paraffin, and cut into 5-μm sections, which were then stained with haematoxylin and eosin (HE), Safranin-O (SO), COL II (monoclonal antibody ab34712, 1:100, Abcam, Cambridge, UK), COL I (monoclonal antibody 600-401-104 S, 1:100, Rockland, PA) and COL X (monoclonal antibody ab49945, 1:100, Abcam, Cambridge, UK), according to our previously established methods^47^,^48^.

### Biomechanical and biochemical evaluations of BEC-vitro

The compressive loading-displacement curve and Young’s modulus of PGA-PLA scaffolds and BEC-vitro at 2, 4, and 8 weeks (conducted by static compression, biomechanical analyser, Instron-5542, Canton, MA, USA, n = 5 for each time point) were recorded and analysed according to a previously establish method^44^. After mechanical testing, samples were collected and minced for quantitative analysis of total GAG^49^, total collagen^49^, COL I, and COL II^44^,^50^. To determine the degradation rate of PGA scaffolds, BMSCs were digested from BMSC-scaffold constructs at various time points (1, 2, 4, 6, 8, 10, and 12 weeks) with papain solution (1:50, Sigma-Aldrich). Next, remnant PGA fibres were collected, dried and weighed (PGA fibres were not digested by papain). Dry weight of remnant PGA fibres were recorded at each time point and plotted to form a PGA degradation curve (n = 3).

### QRT-PCR analysis of BEC-vitro

RNA was extracted from engineered tissues at 2, 4, and 8 weeks, as well as from native cartilage (n = 5). A total of 2 μg of RNA was used to reverse transcribe cDNA according to previously established methods^51^,^52^. QRT-PCR was performed according to the manufacturer’s protocol (Thermo Fisher Scientific). Expression levels of COL IIA1, ACAN, SOX9, COL IA1, COL XA1, and MMP13 were analysed by qPCR using a LightCycler^®^ 480 system with a SYBR^®^ green kit (Roche Molecular Biochemicals, Mannheim, Germany). Beta-actin housekeeping gene was used as an internal control. Forward and reverse primer pairs are listed in Supplementary Table 2.

### Surgical procedures

As shown in Supplementary Fig. 4, two defects with a cylindrical shape (10-mm diameter, 4-mm depth) were created deep into subchondral bone at the weight-bearing area of the femoral medial and lateral condyles of the knee joint. After removal of blood clots, each defect was repaired randomly with either 2-, 4-, or 8-week constructs (two constructs from the same group were stacked together and implanted into the same defect), or left untreated. Constructs were fixed in place by stitching to surrounding native cartilage with biodegradable sutures. Most of the animals (n = 10) were created 4 defects in both knee joints for the implantation of both experimental groups and control groups, while other animals (n = 6) were only created 2 defects in one knee joint for the implantation of 4- and 8-week BEC-vitro.

### Gross observation and grading of the repaired regions

At 6 months post-operation, animals were euthanized to harvest repaired knee joints (distal part of femur). Samples were grossly examined to record surface smoothness, size of the repaired area, and healing interfaces with adjacent native cartilage. Next, samples were sawed sagittally at the midline to observe the healing interface between repaired and adjacent native osteochondral tissues. Gross view results were graded into three scales: complete repair, incomplete repair, and no repair, with criteria similar to a previous report^53^ (Detailed grading criteria were list in Supplementary Table 3).

### Histological examination and grading of repaired regions

Harvested tissues were stained with HE, Safranin-O/Fast green (SO/FG), COL II, COL I, and OCN (monoclonal antibody, 1:100, Abcam, Cambridge, UK) using the same procedure described above. Sirius red (SR) staining under polarized microscopy was also performed to evaluate the distribution of collagen type I according to a previously reported method^54^. To quantitatively evaluate cartilage regeneration, histological grading was performed by three persons through blind analysis, according to previously reported methods^55^,^56^ (Detailed histological grading scheme was list in Supplementary Table 4).

### Biomechanical and biochemical analysis of the repaired tissues

The neocartilage in the reparative cartilage region of different groups (n = 5) were drilled and trimmed into a cylinder shape with a 4-mm diameter, and then used for biomechanical and biochemical analyses using the same procedures described above.

### Statistical analysis

All quantitative data were recorded as mean ± standard deviation. After confirmation of a normal data distribution, one-way analysis of variance and post-hoc least significant difference tests were used to determine statistical significance among the groups. A p-value less than 0.05 was considered statistically significant.

## Conclusion

In summary, the current study demonstrated that i) *in vitro* cartilage formation and maturation using BMSCs represents a time-dependent manner; ii) implantation of BEC-vitro alone can realise tissue-specific repair of osteochondral defects with satisfactory interface integration; iii) the maturity level of BEC-vitro influences repair results. Although several factors that influence repair outcomes still require investigation, the current study provides a promising strategy for future clinical therapy of articular osteochondral defects.

## Additional Information

**How to cite this article**: He, A. *et al*. Repair of osteochondral defects with *in vitro* engineered cartilage based on autologous bone marrow stromal cells in a swine model. *Sci. Rep.* **7**, 40489; doi: 10.1038/srep40489 (2017).

**Publisher's note:** Springer Nature remains neutral with regard to jurisdictional claims in published maps and institutional affiliations.

## Supplementary Material

Supplementary Information

Supplemental Video 1

Supplementary Video 2

Supplementary Video 3

Supplementary Video 4
