# Supplementary material for: Repair of osteochondral defects with in vitro engineered cartilage based on autologous bone marrow stromal cells in a swine model
Source: Sci Rep. 2017 Jan 13;7:40489. doi: 10.1038/srep40489 (PMC5234019; doi:10.1038/srep40489)
Supplement: Supplementary Information [file srep40489-s1.doc]

**Repair of osteochondral defects with *in vitro* engineered cartilage based on autologous bone marrow stromal cells in a swine model**

Aijuan He^1, 2, a^, Lina Liu^1, 2, a^, Xusong Luo^1, 2^, Yu Liu^1, 2^, Yi Liu^1, 2^, Fangjun Liu^3^, Xiaoyun Wang^4,*^, Zhiyong Zhang^1, 2^, Wenjie Zhang^1, 2^, Wei Liu^1, 2^, Yilin Cao^1, 2,*^, Guangdong Zhou^1, 2, 3*^


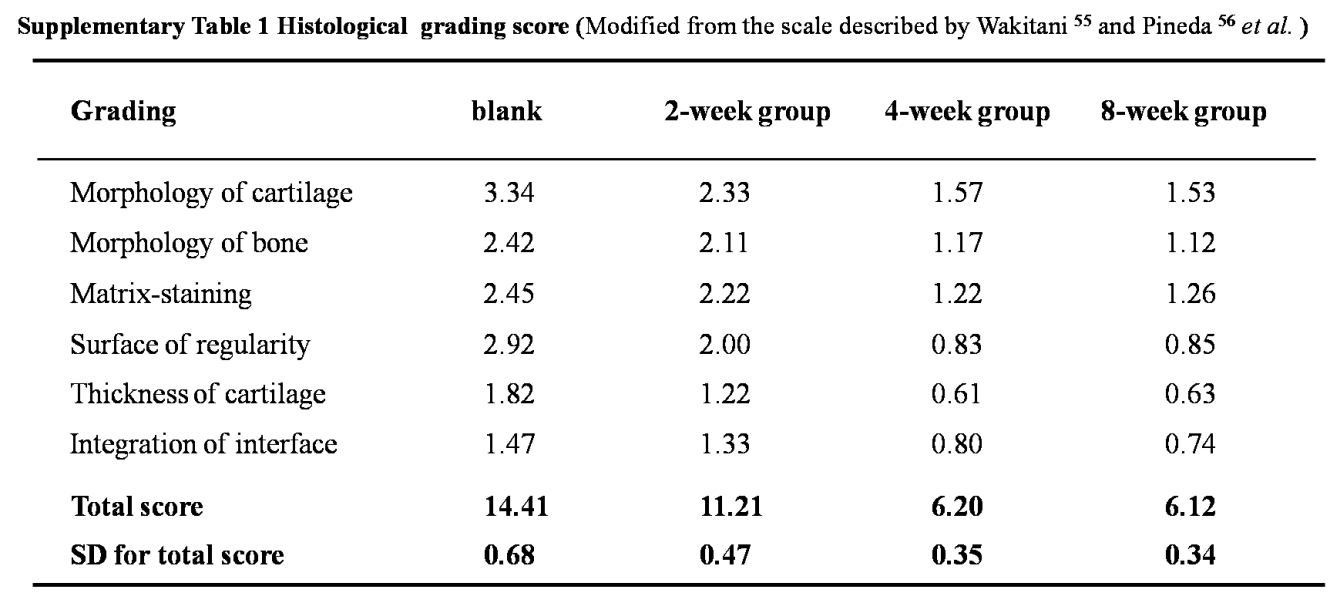


**Supplementary Table 1.** Histological grading score of repaired tissue.


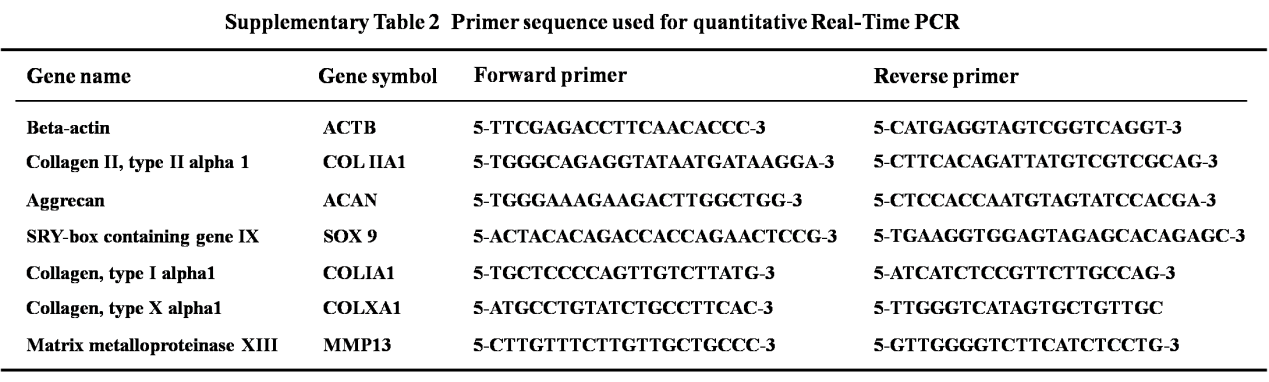


**Supplementary Table 2.** Primer sequences used for QRT-PCR.


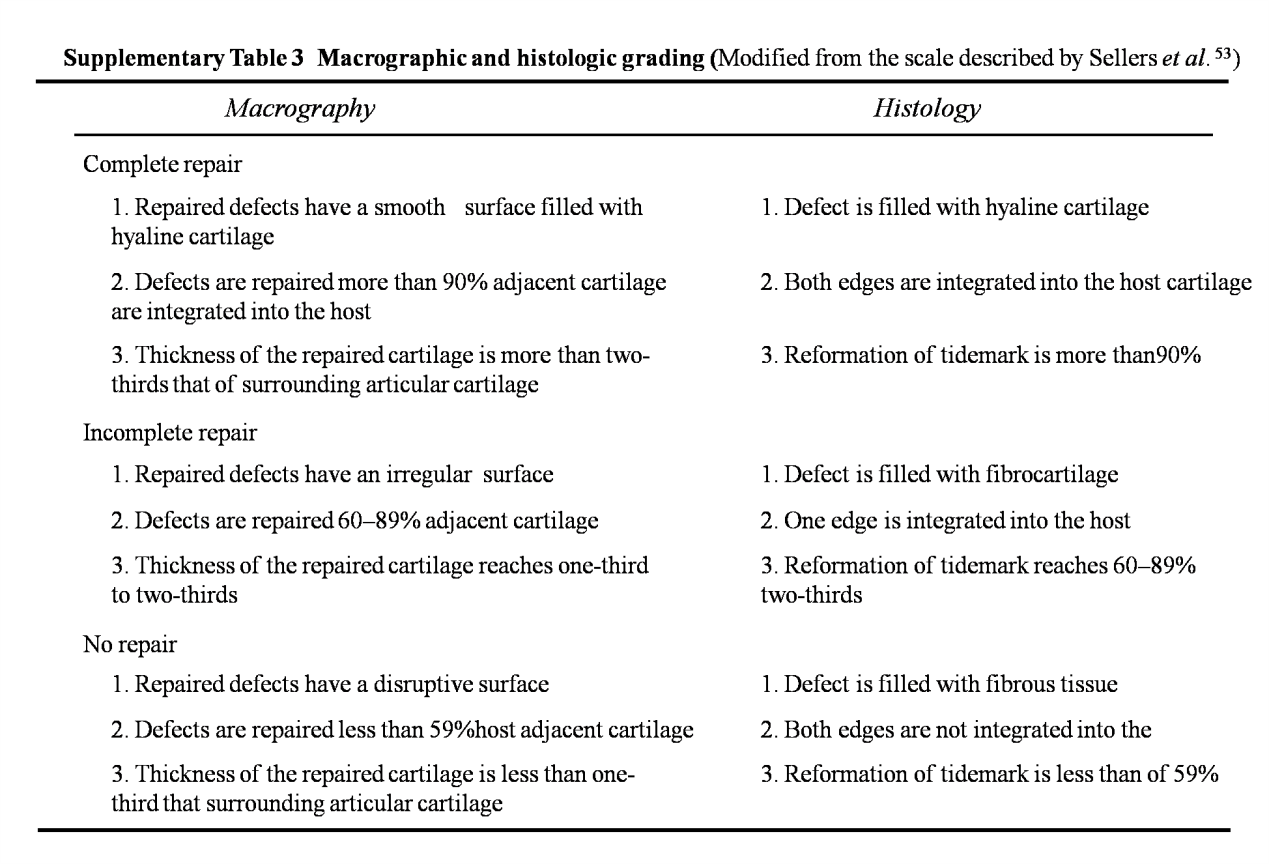


**Supplementary Table 3.** Macrographic and histologic grading for the osteochondral defect repair.


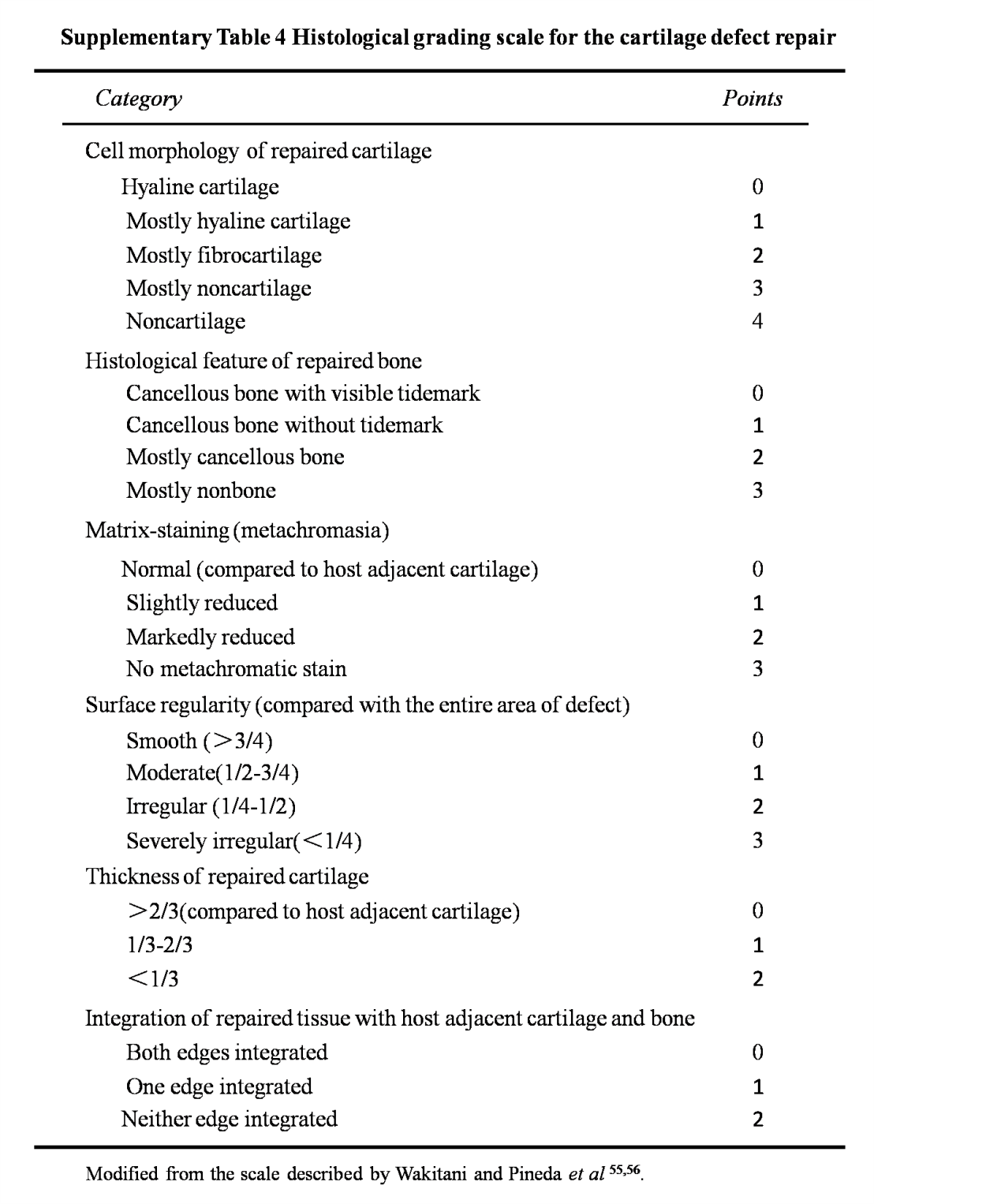


**Supplementary Table 4.** Histological grading scale for the osteochondral defect repair.


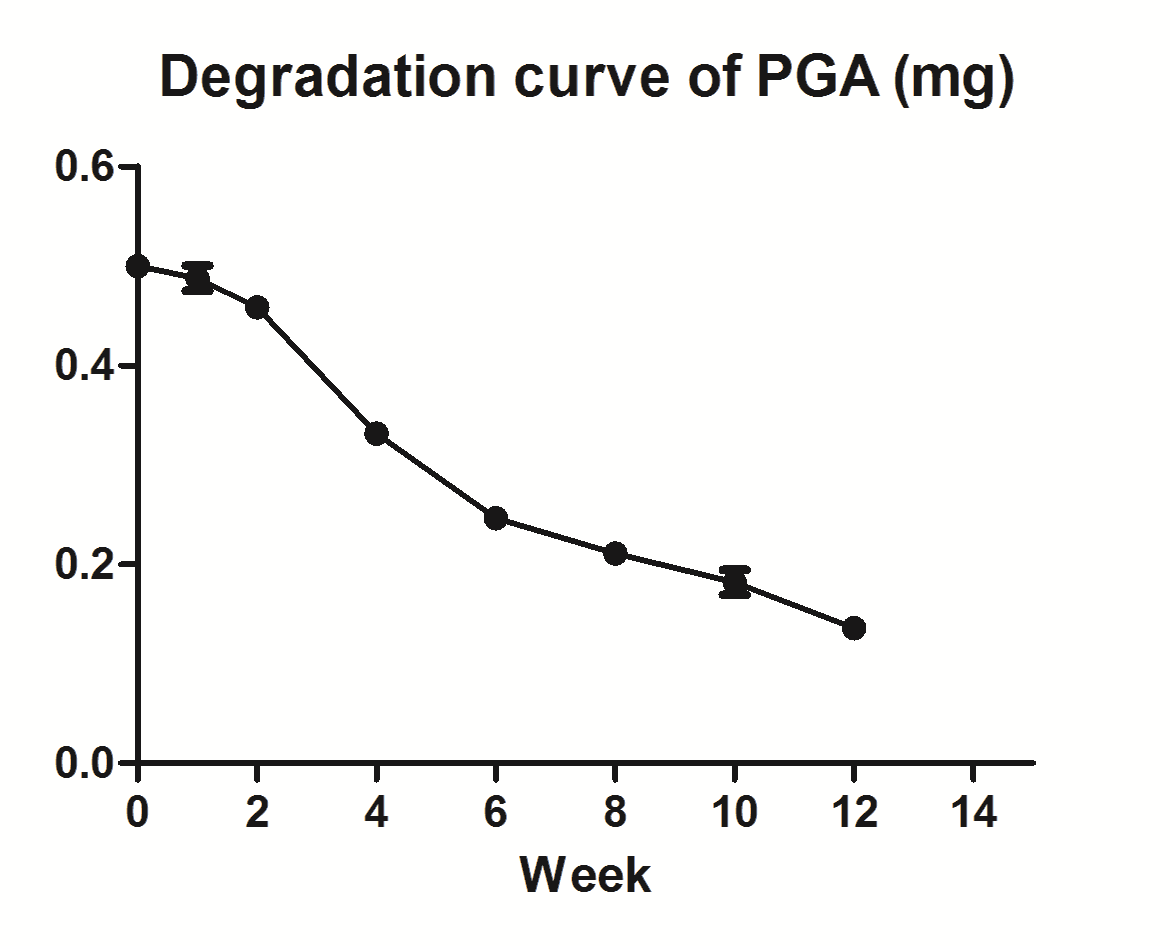


**Supplementary Figure 1.** Degradation rate of PGA scaffolds. PGA fibres showed a decreased trend in wet weight with increased *in vitro* culture time.


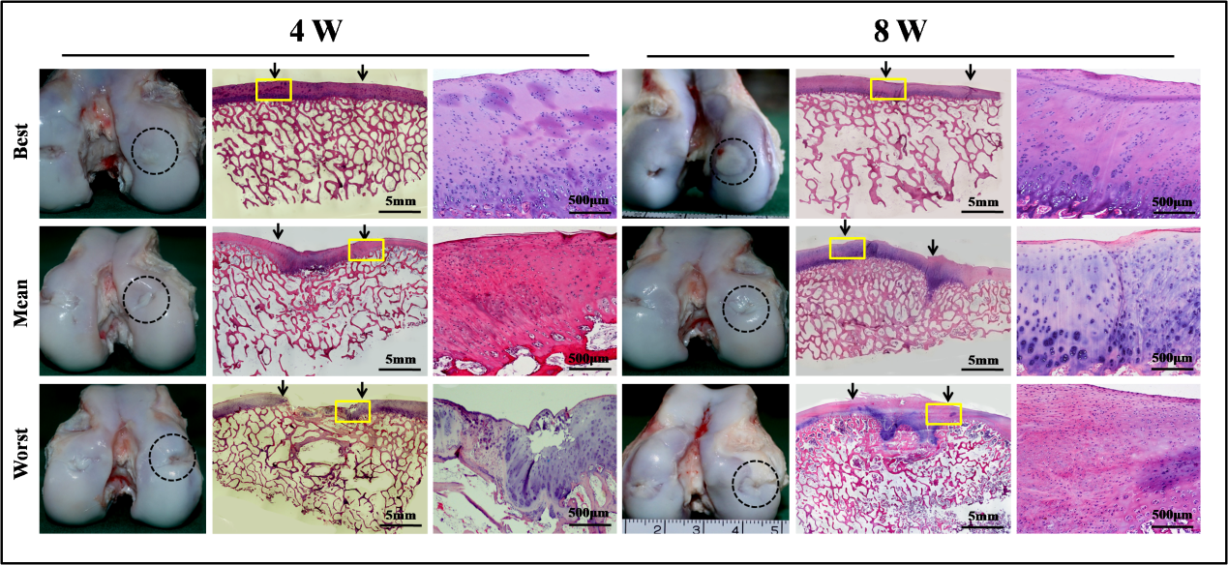


**Supplementary Figure 2.** Gross view and histology of different repair levels in 4- and 8-week groups. The osteochondral defect region at best repair level presents both cartilage-like tissue and bone-like tissue with smooth surfaces, satisfactory interface integration, nearly normal cartilage thickness, and satisfactory subchondral bone regeneration. At the mean level, the defect region is basically repaired by cartilage-like tissue and bone-like tissue, but one or two drawbacks in cartilage thickness and continuity, surface regularity, subchondral regeneration, and interface integration are observed. For the worst level, the defect region presents obvious tissue disrupt, irregular surface, reduced matrix-staining, and incomplete regeneration of both cartilage and subchondral bone. Black rounds and arrows indicate repaired regions.


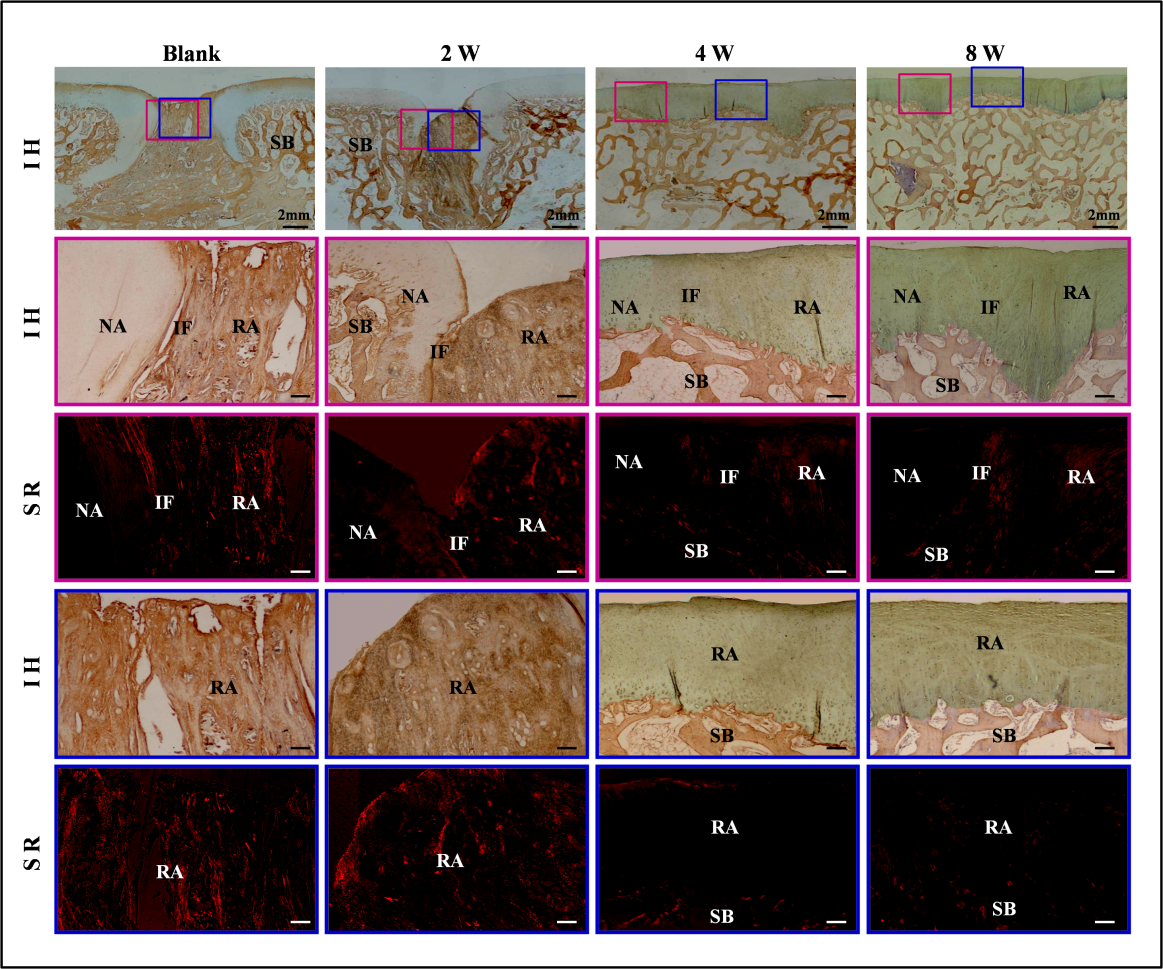


**Supplementary Figure 3.** Examination of collagen I in repaired regions. Strong positive expression of collagen I in IH staining and positive birefringence under polarised light in SR staining were observed in the central areas of repaired tissue in the 2-week and blank groups. In the 4- and 8-week groups, neocartilage close to interface regions showed weakly positive IH staining of collagen I and weak birefringence under polarised light in SR staining; whereas, neocartilage in the central region was basically negative for collagen I expression in both IH and SR staining. High-magnification images in rows 2 and 3 correspond to the pink box region in row 1 of the same group. High-magnification images in rows 4 and 5 correspond to the blue box region in row 1 of the same group. NA: native area; IF: interface; RA: repaired area; SB: subchondral bone; SR: Sirius red staining (polarised microscopy); IH: immunohistochemical staining. Scale bar = 200μm.


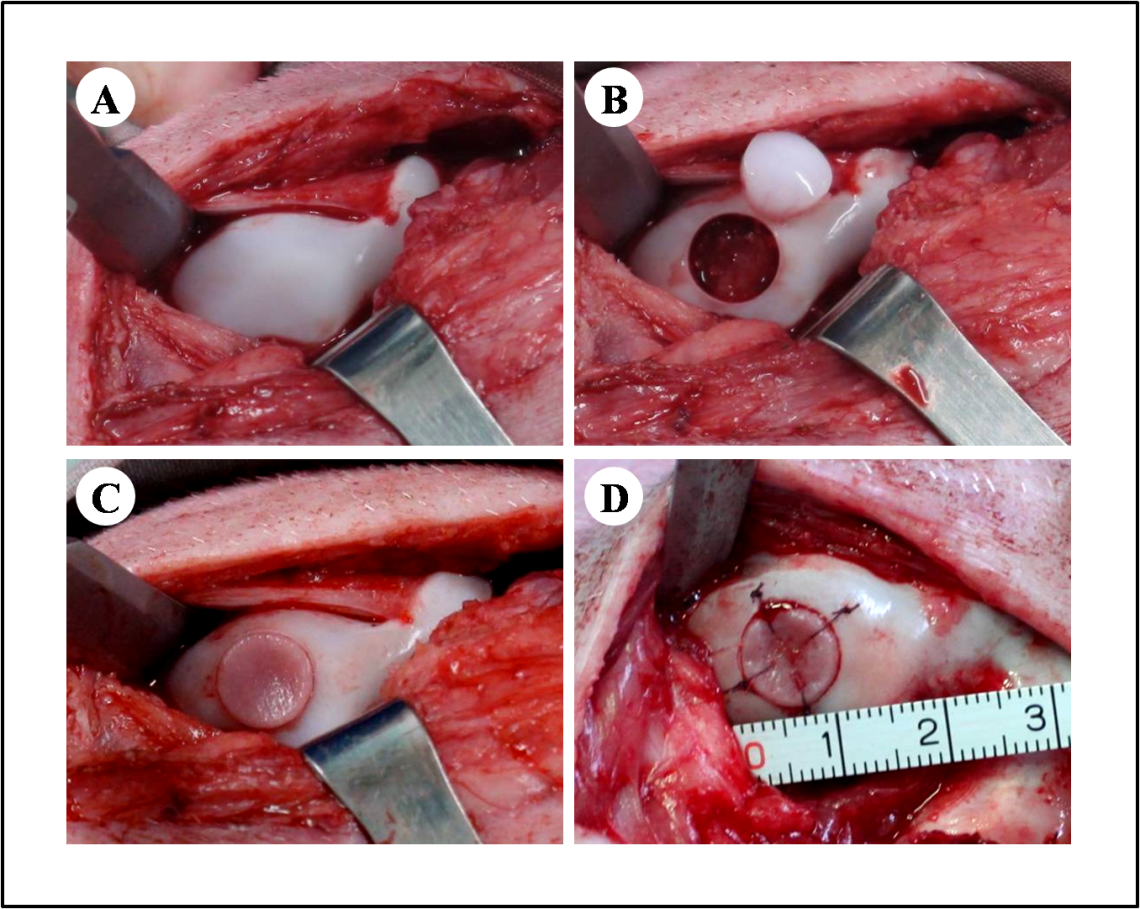


**Supplementary Figure 4.** Surgical procedures. (A) Exposure of femoral condyle. (B) Creation of osteochondral defect. (C) Implantation of in vitro engineered tissue. (D) Sample fixation.

**Supplemental Video 1.** PGA/PLA scaffold.

**Supplementary Video 2.** *In vitro* BMSC engineered cartilage at 2 weeks.

**Supplementary Video 3.** *In vitro* BMSC engineered cartilage at 4 weeks

**Supplementary Video 4.** *In vitro* BMSC engineered cartilage at 8 weeks.
